# Tip-Induced Etching and Vacancy Island Evolution on 2H-TaS_2_ Revealed by STM

**DOI:** 10.1021/acs.jpcc.5c05198

**Published:** 2025-10-08

**Authors:** Dejia Kong, Richard Peckham, Kory Burns, Zhiqiang Mao, Seng Huat Lee, Jordan A. Hachtel, Zheng Gai, Ian Harrison, Petra Reinke

**Affiliations:** † Department of Chemistry, 2358 University of Virginia, Charlottesville, Virginia 22903, United States; ‡ Department of Materials Science and Engineering, University of Virginia, Charlottesville, Virginia 22903, United States; § 2D Crystal Consortium, Materials Research Institute, The Pennsylvania State University, University Park, Pennsylvania 16802, United States; ∥ Department of Physics, The Pennsylvania State University, University Park, Pennsylvania 16802, United States; ⊥ Center for Nanophase Materials Sciences, 6146 Oak Ridge National Laboratory, Oak Ridge, Tennessee 37831, United States

## Abstract

Recent research on 2D materials using scanning probe microscopy reveals that the surface of transition metal dichalcogenides can be etched during the measurement via either a tunneling-field-driven or a scanning probe-driven process. The tip-induced manipulation of the surface structure and defects is a first step toward nanolithography using scanning probes. Real-space scanning tunneling microscopy experiments provide a surface defect inventory, which includes linear and point defects for 2H-TaS_2_ grown by chemical vapor transport. Extended periods of imaging trigger the formation of vacancy islands that grow and coalesce over time, leading to the sequential removal of entire layers. The growth kinetics of vacancies were observed over extended periods and quantified using AI and conventional image analysis tools. The vacancy islands have a linear growth rate of their perimeter and corresponding parabolic growth rates in the area for isolated islands. The growth rate variance of individual islands is discussed in the framework of etching mechanisms including tip-induced chemistry, etching by water adsorbates, and native defects that support vacancy island nucleation in the TaS_2_ surface. New vacancy islands emerge rapidly in newly exposed layers after the top layer is removed. Small and mobile surface islands that are redeposited are revealed to participate in the tip-induced etching mechanism. The quantitative analysis of etching kinetics is a first step toward automated nanostructuring of TaS_2_ surfaces. This work paves the way to use scanning tunneling microscopy to build more complex 2D material structures.

## Graphical abstract

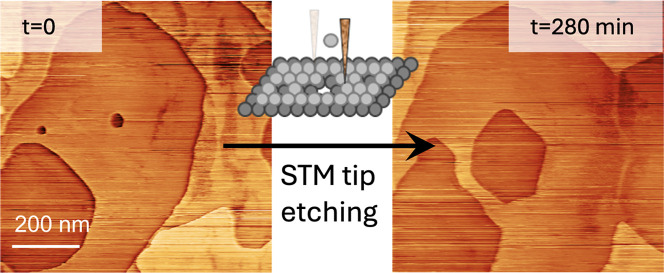

## Introduction

In the pursuit of novel materials, transition metal dichalcogenides (TMDs)^1–3^ have been extensively studied since they show great potential in optoelectronic devices,^4–6^ sensors,^7,8^ and energy storage systems.^9^ In order to make both small and flexible semiconductor devices, a cost-efficient way to produce single atomic layers of TMDs at scale is warranted.^10^ Additionally, methods for isolating, contacting, etching, and integrating TMDs are essential for successful device fabrication. Bulk TMD crystals can be grown using chemical vapor transport (CVT), which is a well-established method in the growth of high-quality materials.^11,12^ All two-dimensional materials, particularly those grown via CVT, often contain point defects formed during the sample growth^5,13^ or postgrowth treatment,^14–16^ which affect the properties of TMDs. While these defects can undermine the durability and consistency of materials, recent theoretical studies suggest that chalcogen vacancies introduce deep in-gap states and can serve as dopants.^17,18^ It is therefore essential to study the relationship between growth conditions and defect formation during growth to identify methods that support optimal device fabrication.

Confined by the van der Waals (vdW) gap between layers, atomically thin TMDs can be exfoliated or removed from bulk TMDs through mechanical contact with adhesive substrates.^10,19,20^ Previous studies have shown that a large-area of single atomic layers of TMDs can be exfoliated by tuning the chalcogen–substrate interaction,^21^ and lateral features of a monolayer of TMDs can be patterned through laser-assisted exfoliation.^22^ These methods are promising in manufacturing single or few layers of TMDs at a large scale, but the performance of TMDs prepared in such a way can suffer compared to bulk materials or intentional single-layer growth. An alternative approach for modifying local surface morphology and introducing patterns required for device integration is based on single-layer etching via scanning probes.^23–29^ This method has the potential to enable direct-write approaches to patterning. It was discovered that the surfaces of TMDs can be etched by scanning tunneling microscopy (STM), and several possible mechanisms were proposed to explain the tunneling current-induced etching.^24^ Similar experiments done with AFM indicate that the participation of a scanning probe is necessary for surface etching, and the water concentration in the experimental environment dictates the etching rate.^25^ An ultrahigh vacuum (UHV)-STM experiment using tip-induced etching on TiSe_2_ indicated that the growth of a vacancy island can be tuned by varying the tunneling current set point, and the etching can be halted by changing the bias voltage.^28^ These studies laid a solid foundation for investigating tip-induced etching, but the kinetics of etching, etch rates, and mechanistic details of the process remain only partially understood, with critical issues warranting targeted study. Further observations of STM-induced etching are needed to provide a deeper understanding of the etching mechanisms in order to achieve superb control over the process. Ideally, tip-induced etching can be used to write device structures or patterns onto TMD surfaces with high precision and yield.

Similar to moving a single atom on the surface, the etching and/or restructuring of the surface can be a result of a combination of several tip–surface interactions including, but not limited to, vdW and electric force between the tip and surface, charge and energy transfer, and bond breaking through electron injection.^30^ In this study, we selected the metallic 2H-TaS_2_ platform to examine STM-induced surface etching and the formation and growth of vacancy islands (VIs). While etching has been reported for the metallic 1T-TaS_2_ phase,^26^ the hexagonal 2H phase, despite being more stable, has not been studied in this context. By investigating the etching mechanisms on the surface of bulk 2H-TaS_2_, our goal is to identify the major contributing factors in large-area tip-induced etching and connect them to intrinsic material properties.

In this Perspective, we begin by assessing the native defects of 2H-TaS_2_ grown by CVT and then study the nucleation and growth of VIs using STM. We identify point defects and a unique set of linear defects near step edges and observe the tip-induced growth of VIs. By tracking the evolution of VIs over several hours, we extract detailed kinetics information on their nucleation and growth. The evolution of the VIs was captured in time-lapse STM topography images, and image analysis of VI characteristics provides quantitative information on growth kinetics. These observations indicate that VI growth kinetics is independent of bias voltage and is likely coupled to native defects in the respective sample section. The interaction volume of the tip and the surface is discussed and local bond breaking and restructuring of the VI edge is proposed as the underlying mechanism for VI evolution. Small segments of TaS_2_ can be moved by the tip and are subsequently reintegrated into the step edge, which is in line with edge restructuring and thus minimization of the edge energy.

## Methods

### Sample Preparation

2H-TaS_2_ single crystals were synthesized by chemical vapor transport (CVT), with iodine as the transport agent. A stoichiometric mixture of Ta and S was transferred into an evacuated 18 cm long, 10 mm inner diameter, and 12 mm outer diameter quartz tube together with 7.5 mg/cm^3^ of iodine. Using liquid nitrogen, the volatile iodine was condensed with the powder at the bottom end of the ampule during the quartz sealing process. To minimize oxide-based Ta growth, all sample preparation was performed in an argon-filled glovebox to reduce the presence of oxygen and moisture. Additionally, the quartz ampule was purged and vented with ultrahigh-purity argon gas to further reduce the oxygen and moisture content prior to sealing the ampule. The sealed ampule was then placed in a four-zone tube furnace and heated up to 950 and 850 °C at the charges zone and growth zone, respectively, for 12 days. The typical lateral size of the resultant flakes was 5 × 5 mm^2^. The X-ray powder diffraction (XRD) pattern of the as-grown single crystal is well-matched with the International Centre for Diffraction Powder Diffraction File (ICDD PDF) card 04-001-0070 and confirmed that the TaS_2_ single crystal is crystallized in the hexagonal crystal structure with the space group of *P*6_3_/*mmc*. Energy-dispersive X-ray (EDX) elemental mapping showed that Ta and S are distributed evenly, and no other elements were detected except carbon and oxygen, whose presence is strongly believed to be from the vacuum chamber and subsequent transport through air. The concentration of the resultant flakes is Ta_1.02_S_1.98_, which is close to the nominal value.

### STM Measurements and Image Processing

The experiments were performed under ultrahigh vacuum (UHV) conditions with a base pressure of 5 × 10^–11^ Torr using a Scienta-Omicron variable temperature scanning tunneling microscope operating at room temperature. The TaS_2_ flake was prepared by mechanical exfoliation in air immediately before being introduced into the load lock chamber (1 × 10^–9^ Torr) and shortly thereafter into the STM chamber. The STM images were acquired in constant current mode at room temperature (297 K). Tungsten tips were prepared by electrochemical etching, where the tungsten wires that are about 5 cm long were spot-wielded to the Omicron tip holder S2701-S, and the wires were submerged into a lamella held by a gold ring electrode. The tips were prepared under UHV on the HOPG surface or on the TaS_2_ surface during the experiment through high-voltage scanning and voltage pulses. STM images were processed with Gwyddion and are plane leveled.^31^ The vacancy island (VI) features were analyzed with two methods: marking the vacancy island perimeter by hand, with subsequent analysis and labeling of the islands with Fiji,^32^ and segmentation with Segment Anything,^33^ and subsequently, area and perimeter were quantified in Fiji.^32^ Both methods yield areas that are within the margin of error and provided near-identical etching rate data.

### Transmission Electron Microscopy

Scanning TEM (STEM) and electron energy loss spectroscopy (EELS) experiments were performed on a Nion aberration-corrected UltraSTEM 100 instrument operated at an accelerating voltage of 60 kV. The experiments were performed with a convergence semiangle of 30 mrad. A 2 mm EELS aperture was used to maximize the analytical signal during acquisition with an EELS collection semiangle of ∼48 mrad. 4D-STEM data was collected with a 2.5 mrad convergence angle.

## Results

STM and scanning transmission electron microscopy (STEM) data on the pristine 2H-TaS_2_ sample are summarized in Figure 1 and confirm the presence of the 2H polymorph. Figure 1a shows a structural model of this phase, and Figure 1b includes an STM image with an atomic resolution. The in-plane lattice constant of 0.34 nm from this image corresponds to the 2H structure unit cell.^34^ An aberration-corrected high-angle annular dark-field STEM (HAADF-STEM) image is displayed in Figure 1c, with a transparent overlay of the molecular modeling image included to emphasize the positions of the atoms in the lattice. In this Z-contrast-based imaging method, the “brighter” atoms represent Ta as they scatter more electrons at higher angles during electron beam interactions, and the S atoms appear dimmer as most electrons scatter at low angles from light elements.^35^ Intensity variations confirm the composition of the film, and the atoms present were also verified qualitatively with electron energy loss spectroscopy (EELS), as described later.

**Figure 1:**
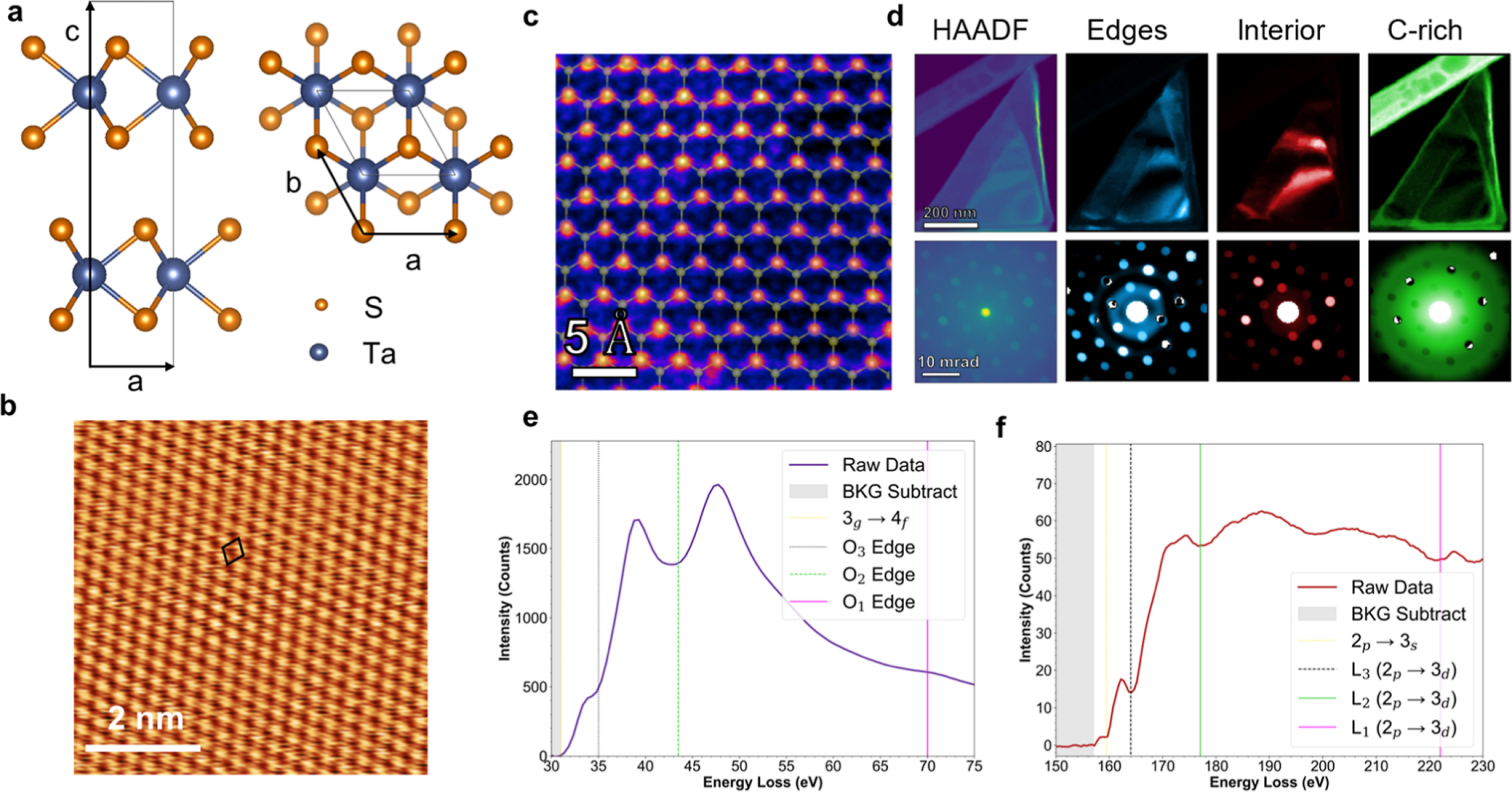
(a) Side view and top-down view of the schematic ball and stick model of 2H-TaS_2_. (b) STM image after 2D FFT filtering showing a parallelogram unit cell of the 2H-TaS_2_ sample. *V*_bias_ = 0.13 V, *I*_t_ = 500 pA. (c) Aberration-corrected STEM image of TaS_2_ showing the short-range order and hexagonal lattice. (d) NMF deconvolution maps of grains in a deposited sheet of TaS_2_ with corresponding nanodiffraction patterns of the selected regions. (e) Ta O-edge and (f) S L-edge confirming elements present in deposited flakes.

Figure 1d presents the 4D-STEM analysis. In this experiment, a converged beam of electrons is rastered across a sample in two-dimensions, thereby creating a two-dimensional diffraction pattern at each location where the beam sits in real space.^36^ To interpret this data set, we used deconvolution mapping in the form of non-negative matrix factorization (NMF).^37^ First, the HAADF micrograph overviews a low-magnification image of the TaS_2_ sheet with, the corresponding averaged diffraction pattern from the spectrum image shown in Figure 1d. Next, since the layer was nonuniform in thickness, which is common in vdW solids,^38,39^ NMF was used to decouple each of the “components” identified in the HAADF overview image into a mask and the averaged diffraction pattern for each region was obtained. The variation between the “Edges” and “Interior” masks of the TaS2 originate from bend contours in the flake, common in large-area 2D materials. The “C-rich” region is masked from drawing analogs to the amorphous rings in the diffraction patterns and shows some carbon accumulation at the edges due to air exposure. The diffraction patterns were all oriented in the same direction, meaning that the films were large single crystals. The intensity ratio of the diffraction peaks corresponds to the subtle variations in the orientation of the crystal with respect to the beam axis. We note the “Interior” mask largely correlates to the area of enhanced thickness identified in the HAADF, indicating that strain connects to the thickness and number of layers in the flake.^40^ In Figure 1e**,** a core-loss EELS spectrum of the Ta O-edge is shown, and while the O-edges are not built for quantification, they are included here to verify the presence of Ta atoms. In Figure 1f, the Sulfur L-edge is displayed, verifying the preference of S atoms in what appears to be S-rich films from the morphology of the dispersed flakes.^41^

Figure 2a shows a large-area STM image, 1000 × 1000 nm^2^, of 2H-TaS_2_, illustrating the general surface morphology of the sample in a region with high defect density. The image includes a monatomic step, and the top layer has the typical triangular shape of transition metal dichalcogenide islands imprinted by the underlying 3-fold crystal symmetry. The topography image is composed of a large-area image on which several higher-resolution images are superimposed. The step edge of the top island shows a high density of linear defects, which are visually reminiscent of fracture cracks. The shape of these linear defects closely resembles the drainage patterns in geomorphology maps. These defects were observed on multiple occasions while surveying large sections of the sample surface and have not been reported previously to the best of our knowledge for any TMD material grown by CVT, CVD (chemical vapor deposition), or MBE (molecular beam epitaxy). The density of the linear defects in Figure 2a decreases with distance from the island and step edges. The longer linear defects are frequently interrupted by “holes” in the top layer, which can undergo branching.

**Figure 2:**
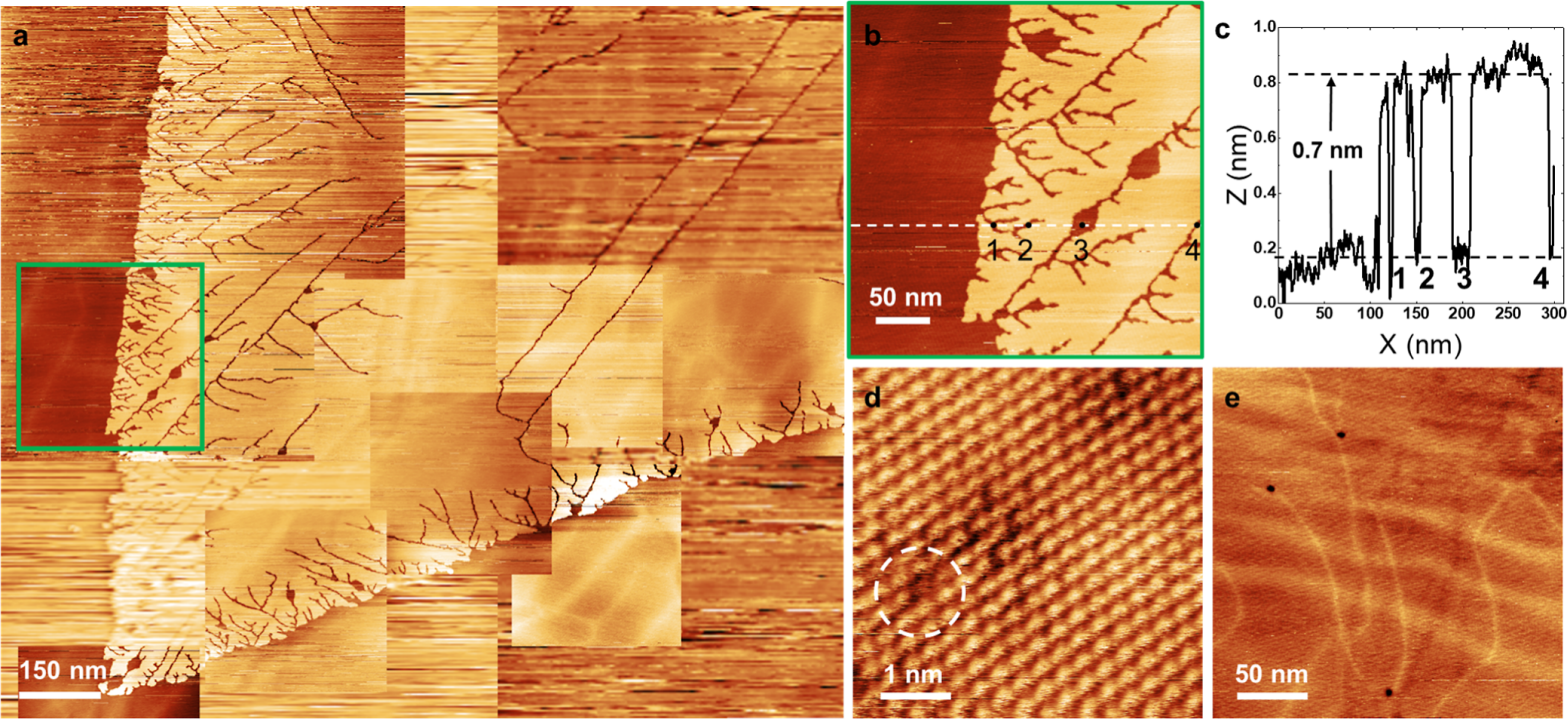
(a) Large-area composite STM image of 2H-TaS_2_ showing the morphology of the surface, with characteristic defects frequently seen around the step edges. (b) STM image with a more detailed view of the linear defects from the region marked by a green square in (a). *V*_bias_ = 1.0 V, *I*_t_ = 20 pA. (c) Line profile taken along the white dashed line in (b). (d) Atomic resolution after 2D FFT filtering to limit noise showing point defects (white dotted circle). *V*_bias_ = 0.1 V, *I*_t_ = 350 pA. (e) Subsurface line defects in lower layers imprinted on the surface. *V*_bias_ = 1.0 V, *I*_t_ = 200 pA.

Figure 2b shows a topography image with a more detailed view of the linear defects, and the line profile was taken along the white dashed line and is included in Figure 2c. Positions 1 to 4 correspond to linear defects, and the height difference across the linear defects is 0.7 nm, which is equivalent to the step height of one S–Ta–S layer. It is worth noting that we did not see any linear defects during the STEM survey, but it is admittedly difficult to find 1D linear defects in a 3D sample with the added challenge that the linear defects might be more prevalent in the near-surface region.

A point defect is visualized at atomic resolution in the white dotted circle and is shown in Figure 2d. The line profile across the defect gives an apparent depth of more than 0.1 nm lower than the signal from the surface atoms. This atomic-scale defect signature might be interpreted either as an S-vacancy or an O-substitutional site due to some similarities between these two types of point defects in semiconducting TMDs.^42^ We currently lean toward the interpretation of a chalcogen vacancy due to the significant height differential, but a reliable interpretation will require a combined STS and DFT study. Figure 2e highlights a segment of Figure 2a where brighter features, which resemble the linear surface defects from Figure 2a in shape and extension, can be directly observed. These features are shallower in apparent height compared to the surface line defects but are very similar in terms of extension across the surface. We interpret them as subsurface line defects that were overgrown during the CVT growth process. The subsequent etching experiments shown in Supporting Information S1, S2, and S3 support this interpretation. Several circular-shaped defects are located mostly at branching points of the subsurface features.

The surface presented in Figure 2 remained stable for more than 24 h of STM imaging, which we tentatively relate to the pristine condition of both tip and sample surface. This particular set of images was acquired immediately after baking the UHV chamber and the tip. In subsequent experiments that followed the introduction of new tips with a transient increase in background pressure, the tip-induced etching and the formation of vacancy islands were observed. Note that this is currently an observation of correlation but not necessarily causation—this is a hint that water or other background gas contributions might be relevant in the etching chemistry. Adsorbates such as water can, even in small concentrations, introduce variations in the electronic and chemical signature of surface defects and alter the tip–surface interactions. Water-induced etching has previously been observed by Wang et al. for MoS_2_ and related to the volatility of transition metal dichalcogenide molecules and fragments^43^ and Delawski and Parkinson, who reported water-related changes in etch rates with AFM.^25^ It is not known whether decorated defects and step edges (O-substitutional defects, O- or OH-decorated vacancies, Ta or S or OH-terminated edges) are more reactive toward etching, or if the adsorption of water on the tip is decisive. However, aside from the presence of adsorbates, the heterogeneity of the sample surface, including variation in defect density, the details of the tip–sample interactions, and imaging conditions, has to be examined. In the current experiment, we remain mostly blind to the details of the defect inventory at the atomic scale when the sample is first introduced. Specifically, information on defect type, decoration with adsorbates, or lack thereof and the contributions of adsorbates on the basal plane are not yet available since we chose to focus on the larger length scale.

It was observed that surface etching and growth of VIs can be induced by scanning and imaging with an STM tip on 2H-TaS_2_. We describe here the emergence of VIs, their coalescence and growth, and the removal of entire TaS_2_ layers through tip-induced etching. A quantitative analysis of the images, as described in the Methods section, is used to determine the growth rates of individual, isolated VIs, and coalescence events. An extended set of image stacks illustrating various scenarios of VI formation is included in the Supporting Information and referenced throughout the manuscript.

Here, we present data and analysis for images with sufficient quality to distinguish VI boundaries and perform feature extraction for quantitative analysis. The tip was scanned over an area of 800 × 800 nm^2^ for more than 48 h in total. Five layers of TaS_2_ were removed within the scanned area over this period of time, and removal occurred by nucleation, growth, and coalescence of VIs—an inverse of the growth process. Due to the constant change of the tip state in this process, the images tend to be rather streaky, which is a substantial challenge for tracking individual features, especially at the beginning of the experiment when many VIs were nucleating simultaneously. This is seen in the GIF stacks of some of the etch sequences, which are included in the Supporting Information Figure S1-3 and Figure S4-1–S4-3. For quantitative analysis of VI growth kinetics, 8 VIs were selected, which are well isolated from other islands and did not undergo coalescence within the accounted data range and whose images included a sufficient number of frames with consistent image quality for quantitative feature analysis. One VI that undergoes coalescence is included in the analysis. The process for measuring the VI dimensions is described in the Supporting Information Section S5 and Figure S5.

Figure 3a and b show an example of an isolated vacancy island (designated as VI#4 in Figure 3b) at the center of the images and selected for the study of its growth kinetics. All selected isolated islands were tracked, and their area and perimeter progression are plotted against time in Figure 3c and d. The perimeter data were fitted with linear functions and yielded an *R* ^2^ > 0.99, and the slope represents the VI growth rate. Perimeter and area are given in units of pixels, where one pixel corresponds to 1.56 and 2.44 nm^2^, respectively. The perimeter growth rates vary by about a factor of 5, and it remains challenging to determine unequivocally the correlation between rate, materials characteristics, adsorbate, and defect density in the surface.

**Figure 3:**
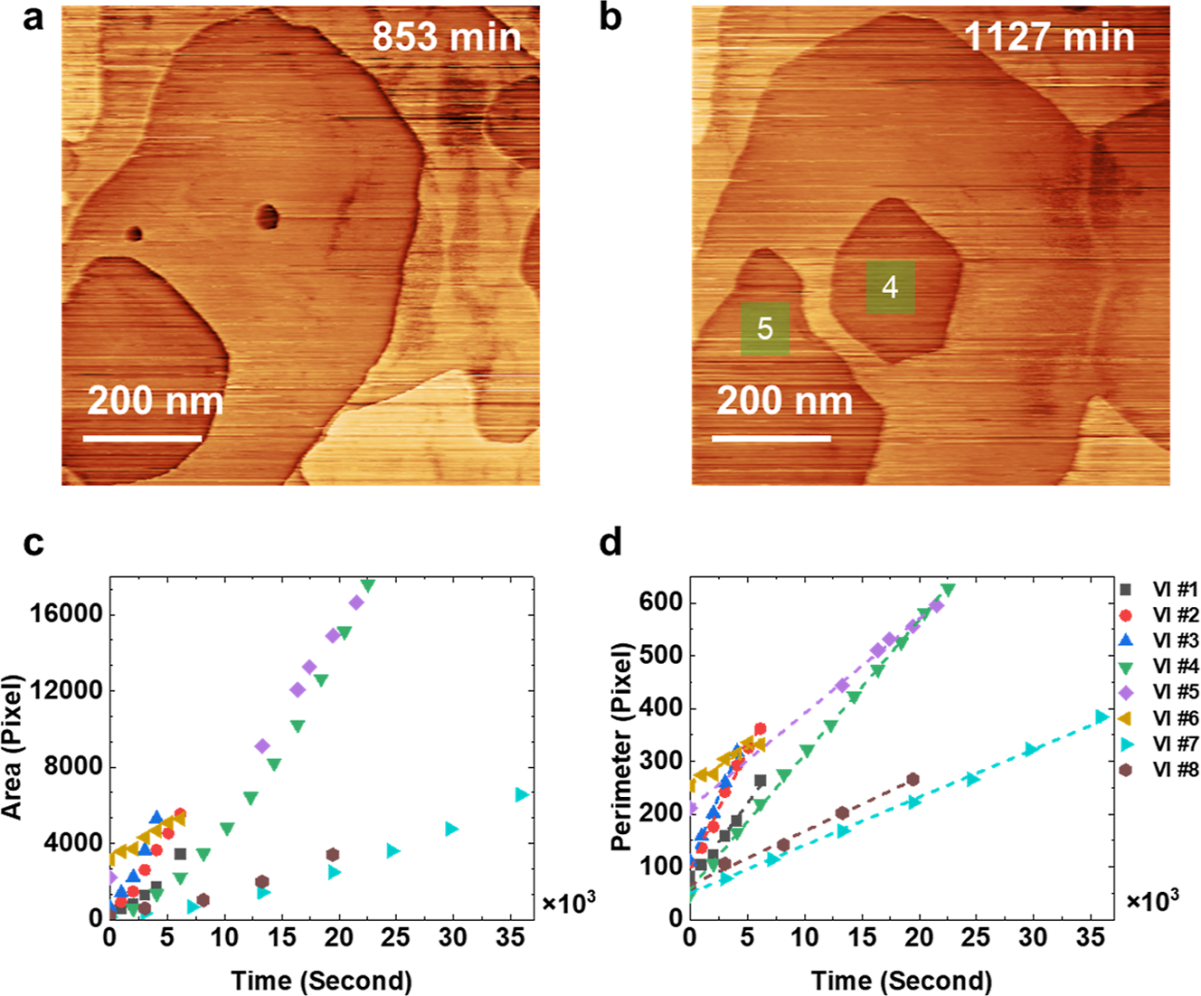
(a,b) STM images of an emerging vacancy island at different times of scanning. This is VI#4 seen in the center of the image. These two images were recorded 244 min apart. *V*_bias_ = 1.5 V, *I*_t_ = 20 pA. Scanning speed = 1600 nm/s. (c) Area for 8 different isolated vacancy islands as a function of scan time. (d) Perimeter progression and linear fits (dashed lines) of the same 8 vacancy islands.

The growth rates vary among VIs, but they are nearly insensitive to the sample bias voltage, for example, between 0.5 and 1.5 V for VI#4. Changes in bias voltage during the growth of specific VIs are shown in Figure 4 and summarized in Tables S1 and S2 Supporting Information. The variation in bias voltage does not correlate with the VI growth rate when comparing different VIs. We restricted the variation in imaging conditions to a relatively narrow range close to commonly used imaging conditions to limit the number of variables. In the current work, we establish a baseline for variability in etching kinetics, which still leaves significant variation in rate. Future work will extend toward both lower and higher values of bias voltage and tunneling current to probe threshold values for tip-induced reactions.

**Figure 4:**
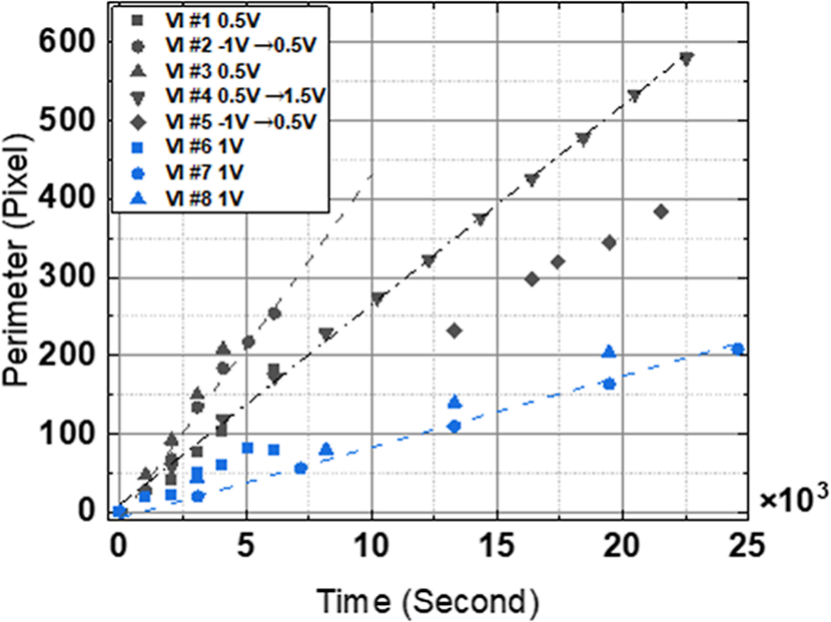
Perimeter progression versus growth time for VIs with the legend that indicates vacancy island number and bias voltage conditions. The tunneling current set point for black data points is 20 pA and for blue data points is 50 pA.

The tunneling current was increased during the growth of VI#6–8 from 20 to 50 pA, and this reduction appears to lead to a decrease in the etch rate, which is less for VI#6 than for VI#7 and 8. This is not intuitive since a higher etch rate is generally expected for smaller tip–surface distances (higher feedback current with the same bias voltage of 1.0 V) and consequently increased local electric fields. Alternatively, the concentration of defects that serve as VI nucleation points might vary within the surface and between top and lower layer and thus introduces an additional control parameter. VI#6–8 are indeed positioned 5 layers deeper than the native surface, and as such, their somewhat lower etch rate can be related to materials variability rather than imaging parameters.

In addition, the growth rate of the VIs is independent of their size, as seen in the linear (parabolic) increase in perimeter (area) as seen in Figure 3c and d. This means that the initial size measured for the first image of each VI also does not correlate with the VI growth rate: for instance, VI#6 has a growth rate similar to those of VIs #4, #8, and #7, despite beginning with a much larger initial area (VI#6, VI#7, and VI#8 images in the Supporting Information Figures S4-2 and S4-3).

As the area of a VI increases, the hexagonal shape becomes more apparent (see Figure 3b). Since 2H-TaS_2_ is in the *P*6_3_/*mmc* space group, the hexagonal vacancy island shape is indeed expected and minimizes the boundary energy of the perimeter. The thermodynamically preferred hexagonal shape may be disrupted during coalescence events or in the vicinity of defect sites, which pin the VI boundary, and is seen in Figures 5 and 6 and Supporting Information Figure S3. This leads to a wide range of VI shapes, and the VIs will eventually merge; finally, a complete layer will be removed.

**Figure 5:**
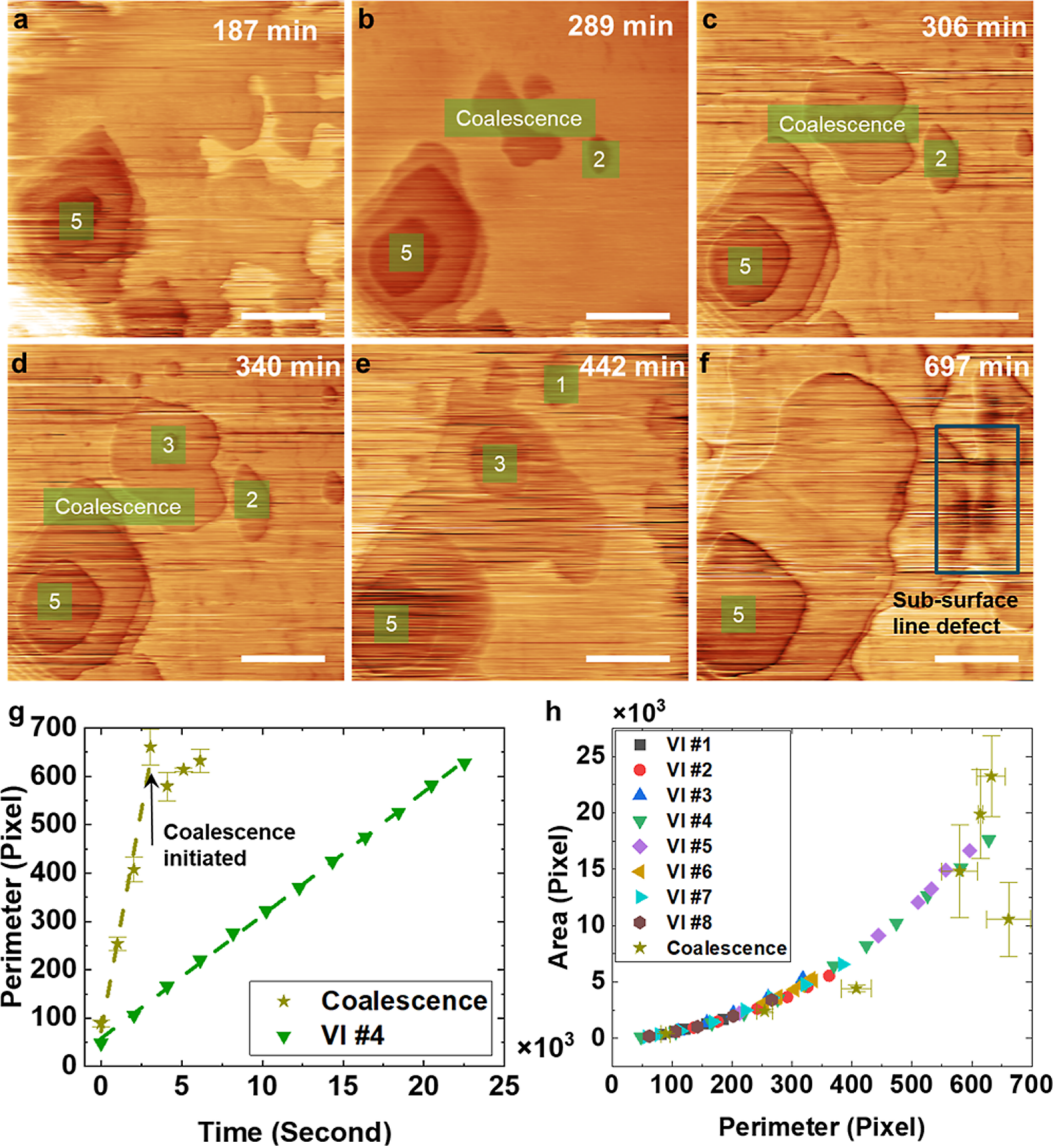
(a–f) Sequence of STM images recorded for the same scan area with initially several individual vacancy islands, which coalesce over time. Time stamps on the images indicate time since the start of image collection. *V*_bias_ = 0.5 V for (b–f) and −1 V for (a), *I*_t_ = 20 pA. Scanning speed = 1600 nm/s. Scale bar = 200 nm. The vacancy islands that were extracted are labeled, and the numbers correspond to the labels in area and perimeter graphs used also in Figure 3. (g) Comparison between the total perimeter over time for a VI undergoing coalescence and the isolated island #4. (h) Area versus perimeter for all isolated vacancy islands.

**Figure 6:**
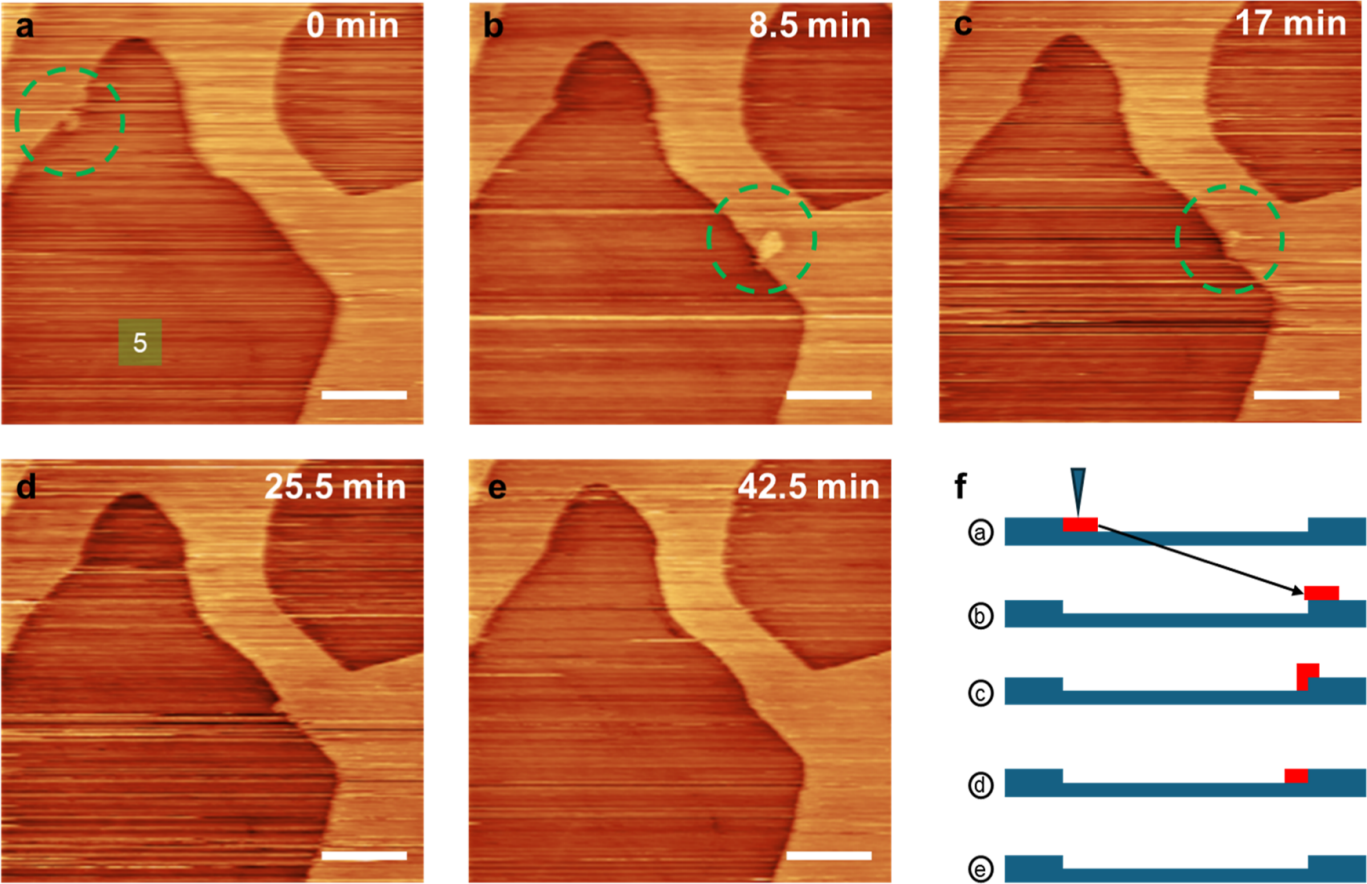
(a–e) Sequential STM images showing the movement of a dislodged 2H-TaS_2_ island, which is moved by the STM tip and recombines with the layer at the step edge. Scale bar = 50 nm. (f) Schematic representation of the moving island (red) and the two stationary layers of TaS_2_ (blue). *V*_bias_ = 1.5 V, *I*_t_ = 20 pA. Scanning speed = 1600 nm/s.

Figure 5 illustrates the growth and coalescence of VIs in a single set of images. Islands VI#3 and VI#2 in Figure 5a–f undergo coalescence and were included in the analysis of isolated islands (Note: Figures 3 and 5 include the same experimental series and the numbers refer to identical islands). In Figure 5a–f, a series of STM topography images featuring multiple coalescence events is presented. Two batches of islands in Figure 5a–f stem from similar locations across multiple layers: one being roughly the center of the image where coalescence happened first and the other at the bottom left corner labeled VI#5, which eventually coalesces with (VI#2 + VI#3). VI#5 was originally “stacked” three layers deep and resulted from a previous series of scans observing VI evolution in the vicinity of closely aggregated step edges (Supporting Information Figure S1). In addition, a subsurface linear defect is uncovered at the end of this series (*t* = 679 min) on the right-hand side of the image in Figure 5f. The pinning of the recession of the TaS_2_ layer at this subsurface line defect is shown in more detail in Figure S3 of the Supporting Information.

The area and perimeter progression for a coalescence event between VI#2 and VI#3, as well as the growth of several isolated islands, are plotted in Figure 5g and h. The error bars included for the “coalescence” island are significantly larger than the rest of the data points due to the uncertainty generated by the coalescence events and the lack of sharpness of the perimeter. The isolated VIs area and perimeter follow their set geometric relation, and all data sets collapse onto a single curve in Figure 5h—coalescence events are easily identified since they do not follow the same geometric relation. In the case of coalescence, the VI area continues to increase but the perimeter remains nearly constant. This behavior is consistent with the minimization of the line energy, represented by the perimeter length as the driving force for coalescence. The area vs perimeter plot in Figure 5f also shows that VI#2 and VI#3 follow the trends appropriate for isolated islands before coalescence. But before the coalesced island VI#2 + VI#3 can reach the optimal hexagonal shape that would further reduce the line energy, it coalesces with VI#5 and several other small VIs, resulting in the near-complete removal of a layer within the scan area. This is different from Delawski and Parkinson’s observations on SnSe_2_ with AFM tip-induced etching, which present a nonlinear increase in the perimeter but the area of the VIs goes through a maximum.^25^ The number of coalescence events in their study is larger than in our case and caused by a significantly higher density of pit-forming events. In contrast, our work shows a sparse pit nucleation density and concomitantly fewer coalescence events. Hence, the VI growth where perimeter and area are related by the geometric formula of a hexagon dominates for many VIs. Note that on some areas of the sample, rapid pit growth is seen and paired with frequent coalescence events extending over several layers of TaS_2_, which does not allow for a reliable quantification of growth kinetics.

In addition to the etching events, it is also observed that small TaS_2_ islands can break off from the large “continent” on the same layer and be redeposited on another section of the layer. These small islands become mobile and can be moved by a scanning probe. This is seen in Figure 6 where a small TaS_2_ island (∼300 nm^2^) was detached from one step edge and relocated to the top of step edge on the right of VI#5. A cartoon illustrating the relative vertical position of the moving island (red) with respect to the stationary layers of TaS_2_ (blue) is shown in Figure 6f. In Figure 6a, a small island (green dotted circle) is observed attached to the top left side of the vacancy island; after several scans; in Figure 6b, the island is moved on top of the step edge on the right side of the vacancy island. From Figure 6c to e: in Figure 6e, the “mobile” island gradually merges with the underlying step edge, now appearing as an additional “bump”, and is fully integrated in the previously straight edge without height variation. The seamless recombination indicates that the island is in fact a TaS_2_ island instead of surface impurities or other byproducts from the etching. This shows another type of tip-induced reaction, where a nanometer-sized piece is moved across the surface and reverses tip-induced etching locally. It is worth noting that the time frame for this process is shorter than the interval between time marks on the images since only a small portion of time contributes to the moving of the island (less than a tenth of the total image scan time). It is particularly interesting to see that the nanosized island is fully integrated in the edge of the VI in Figure 6e, which indicates a significant driving force toward edge faceting and mobility of TaS_2_ molecules or fragments. In addition, while we see in these images the motion of a nanoscale piece of material, one may conjecture that smaller fragments can also be moved through the tip interaction. Hence not only does the electron-induced bond breaking play a role in the surface dynamics of VI nucleation and growth, but tip-induced surface diffusion also contributes to the process.

## Discussion

The interaction between the tip and the surface can lead to the nucleation of VIs, which undergo growth and coalescence events during continued scanning of the surface. The shape of isolated VIs is hexagonal they can adopt various geometries after undergoing coalescence. The edges of VIs restructure over time, gradually approaching the preferred hexagonal edge termination, as dictated by the underlying hexagonal lattice. The emergence of triangular or hexagonal VIs and etch pits has been described for several TMD materials,^25,28,43^ and our work is in agreement with these reports. The dominance of hexagonal VIs indicates that etch rates are independent of the edge termination of the TMD facets. The edge refaceting is particularly evident in Figure 6, where a small TMD fragment dislodged by the tip is incorporated seamlessly in the step edge after several imaging cycles. The significant “streaking” visible in the images during scanning is an additional indication of the rapid and facile diffusion of TaS_2_ of (TaS_2_)_n_ fragments on the surface. Over time, the VI growth leads to the removal of complete TaS_2_ layers within the scan area, while the region outside the scan window remains unchanged (Supporting Information Figure S2). This finding is important for controlling the patterning of TaS_2_ in nanoscale device fabrication and processing.

The VIs tend to nucleate at defects such as line defects, which are prominently featured in Figure 2 and in S7 (Section S7) in the Supporting Information or at multilayer steps shown in Figure S1 in the Supporting Information. It is very likely that many VIs originate at point defects or defect clusters on the surface, albeit the larger scale images do not provide direct visual confirmation. The growth rate was determined for isolated islands, with a total of 8 VIs examined during different scanning stages. The number of islands amenable to this analysis is relatively small since coalescence is a frequent event. Coalescence leads to a rapid increase in area while (nearly) conserving the perimeter length in accordance with an Ostwald ripening mechanism. Initially, the perimeter of coalesced islands is not faceted, and faceting of the step edges to minimize line energy occurs on a longer time frame. Note that “rearrangement” here can be achieved by local detachment or attachment of molecules that are mobile on the surface.

Each pixel in an 800 × 800 nm^2^ image corresponds to 2.44 nm^2^ and 25 unit cells of 2H-TaS_2_, whereas each side of the pixel with a length of 1.556 nm is occupied by 4.7 unit cells. To achieve an increase in perimeter at a rate recorded for VI#4 (Figures 2 and 3), about 40 pixels corresponding to about 200 unit cells must be removed from the entire perimeter per pass with the STM tip (or per image). This calculation does not include detachment–diffusion–redeposition rates. Interestingly, the rate of VI growth does not depend on the initial VI size, no clear correlation between rate and imaging conditions can be established, and no directional dependence of the etch rate with respect to slow and fast scan directions or edge termination is evident. We propose therefore that the etching itself is initially a local event where a TaS_2_ molecule or a fraction of such a molecule is removed from the perimeter of a VI through the initiation of a local bond breaking, most likely triggered by the tunneling electron and possibly facilitated by defects and adsorbates. The presence of defects promotes the nucleation of VIs and the diffusion of TaS_2_ molecules or fragments aids in this process. This implies that the condition of the sample surface, the material heterogeneity, and the presence of adsorbates are relevant and the basis of tip-induced surface chemistry. This model is consistent with our assessment of the rate of removal of molecules from the VI perimeter, consistent with prior observations by Delawski and Parkinson^24,25^ and supported by a strong dependence of tip-induced etch rates on the choice of the TMD material.

However, the variability in etch rates between isolated VIs quantified in Figure 3 remains an open question. There is a high sensitivity of the process to relatively small variations in “vacuum quality”. Our very first scans of the surface, which were performed immediately after a chamber bakeout that included the tip, showed a rather stable surface, and VIs did not develop over several hours of imaging (i.e., Figure 2). Subsequent images presented here were recorded at later times, after the tip needed to be exchanged, and despite the use of a load-lock to introduce a new tip, minute amounts of water are likely to have entered the chamber. We propose that water adsorbates, even in small amounts, might contribute to accelerating etch rates albeit the detailed mechanisms have not been resolved. It has been reported that under ambient conditions, the tip-induced etching observed with AFM indicates elevated humidity can contribute to the acceleration of etching rate.^25^ It is also known for other systems that small amounts of adsorbates can dramatically change growth and reaction pathways.^44^ Specifically for TMDs, the reaction of the step edge with water is rapid and has been used to “carve” triangular defects and even large trenches into MoS_2_.^43^ Another scenario is that the density of defects, or VI nucleation sites, may vary across the surface and contribute to the local variability in etch rates. This can only be resolved if we manage to record the defect inventory without triggering VI nucleation. Finally, cross-talk coupling between adjacent VIs, terraces, and features on the surface can control the concentration of molecules on the surface by acting as sources or sinks. This in turn controls not primarily the etch rate of the tip but the recombination rate at the perimeter. In general, diffusion lengths on 2D material surfaces are relatively long on terraces, and such crosstalk can extend far beyond the imaging range captured by the STM. The pinning of the VI perimeter at a subsurface line defect shown in the Supporting Information Figure S3 might be a consequence of a locally increased recombination rate. It is not known whether etched molecules can desorb from the surface or, alternatively, remain on the surface, forming what might be described as a “2D molecular gas”.

Each of these processes can be tested individually, and further experiments and theories will lead to a more complete quantitative description of the etching process. This is needed to establish tip-induced etching as a process suitable for deterministic pattern writing to build TMD heterostructures and to achieve integration with other processes on the path toward 2D materials-based device architecture.

## Conclusions

In conclusion, our work demonstrates the feasibility of the tip-induced etching of TaS_2_ as a method of nanostructuring. Our study reveals unique intrinsic defects on the surfaces of CVT-grown 2H-TaS_2_ materials. The linear defects are associated with subsurface structures, which suggests that the CVT growth process can lead to clustering and propagation of defects. The tip-induced nucleation and growth kinetics of VIs are quantified using AI-assisted thresholding methods. The growth kinetics of VIs is discussed, and several mechanisms and reaction pathways are proposed. It is suggested that minute amounts of water and surface defects might be most significant for VI growth kinetics and contribute to tip-induced local chemistry. In addition, significant crosstalk between adjacent VIs likely exists due to long diffusion paths of TaS_2_ molecules and fragments and modulates the VI growth kinetics. Our findings reveal key elements to fast tip-induced etching that holds promise for 2D material manipulation using STM. The objective is ultimately to program the STM tip motion to write complex features into the TaS_2_ surface, akin to nanoscale lithography. Unlike chemical or ion-induced etching, which is prone to irretrievably damaging the TMD material, the tip-induced etching promises a more gentle but tightly controllable approach. The current manuscript indicates the feasibility of tip-induced lithography, but further UHV studies on etching kinetics with controlled water ambient pressure, surface defect inventory, and a wider range of scanning parameters (bias voltage and tunneling current) have to be conducted in order to establish a kinetic model for the etching as a prerequisite for programmability of feature writing.

## Supplementary Material











## Data Availability

The data sets that support the findings in this study are available from the corresponding author upon request. This data package contains information on the growth conditions and characterization results of TaS_2_ bulk single crystals used in the study of the tip-induced etching and vacancy island evolution revealed by scanning tunneling microscopy. The TaS_2_ bulk single crystals were grown using the chemical vapor transport (CVT) technique at the NSF 2D Crystal Consortium facility. The package includes sample preparation, recipe file, as well as SEM and XRD data files, and information about the four-zone tube furnace used in this workLee, Seng Huat (2025). TaS_2_ Bulk Single Crystal for Scanning Tunneling Microscopy [Data set]. Scholarsphere. https://doi.org/10.26207/q33z-eb56.

## Notes

Figures S1-1 to S1-3: Stacked VIs and bunched steps generated by STM etching with discussion of time sequence and drift including a GIF stack of the etching sequence. Figure S2 shows a test confirming the participation of the scanning probe tip in the etching process through variation in the size of the imaged region. Figure S3 illustrates the pinning of VIs at subsurface features. Figure S4-1 includes a GIF stack of etching along a steep edge with multiple layers, and subsequent emergence of isolated VI, their growth and coalescence on a large terrace. Figure S4-2 includes a GIF stack which illustrates the evolution of several islands, and confirms the pinning by sub-surface linear defects. Includes Tables S1 and S2 with imaging information. Figure S5 illustrates the process used to measure VI dimensions from STM images. Figure S6 shows the STM image of a screw dislocation. Figure S7 illustrates the effects of extended etching periods and includes a GIF stack recorded over an extended etching periodwith a more detailed view of layer removal. All GIF stacks are attached as MP4 formatted videos to accompany he supplemental text.(PDF)
Figures S1 to S3-1 (MP4)
Figure S4-1 (MP4)
Figure S4-2 (MP4)
Figure S7 (MP4)

## #

Physical Sciences Division, Pacific Northwest National Laboratory (PNNL), Richland, Washington 99352, United States
