# Supplementary material for: Tip-Induced Etching and Vacancy Island Evolution on 2H-TaS2 Revealed by STM
Source: J Phys Chem C Nanomater Interfaces. 2025 Oct 8;129(42):19166–76. doi: 10.1021/acs.jpcc.5c05198 (PMC12557395; doi:10.1021/acs.jpcc.5c05198)
Supplement: Supplementary file 1 [file jp5c05198_si_001.pdf]

Supporting Information

Tip-Induced Etching and Vacancy Island Evolution on 2H-TaS_2 Revealed by STM

Dejia Kong [1,†,*], Richard Peckham [1], Kory Burns [2], Zhiqiang Mao [3,4], Seng Huat Lee [3,4], Jordan A. Hachtel [5], Zheng Gai [5], Ian Harrison [1], Petra Reinke [2,*]

[1] Department of Chemistry, University of Virginia, Charlottesville, VA 22903, USA

[2] Department of Materials Science and Engineering, University of Virginia, Charlottesville, VA 22903, USA

[3] 2D Crystal Consortium, Materials Research Institute, The Pennsylvania State University, University Park, Pennsylvania 16802, USA

[4] Department of Physics, The Pennsylvania State University, University Park, Pennsylvania 16802, USA

[5] Center for Nanophase Materials Sciences, Oak Ridge National Laboratory, Oak Ridge, TN 37831, USA

[†] Current address: Physical Sciences Division, Pacific Northwest National Laboratory (PNNL), Richland, WA 99352, USA.

[*] Corresponding authors: pr6e@virginia.edu, dk7cw@virginia.edu

S1 Stacked vacancy islands (VIs) and bunched steps generated by STM etching

This section illustrates the etching process across a larger image frame and discusses the impact of switching frame size and drift. At the initial stage of the experiment, after rapid etching of the surface material was first observed, we interrupted the scanning and reduced the image frame size from 800×800 nm^2 to 200×200 nm^2, scanned at the reduced frame size for about 3.5 hours, and finally switched back to 800×800 nm^2. The comparison between the images before and after the switch, plus the images collected at 200×200 nm^2 are shown in Figures S1-1 and S1-2, and S1-3 which includes a GIF movie set up from a complete STM image set to visualize the surface evolution over time. A square of etched area that corresponds to the 200×200 nm^2 frame is marked in Figure S1-1(b) which was recorded after returning to the larger image size. The image drift for this experiment was about 0.038 nm/s and is indicated by the green arrow in Figure S1-1(a), and was exacerbated by switching image dimensions. Therefore the smaller image square marked in Figure S1-1(a) is not at the center of the frame.

Figure S1-1 includes the 800×800 nm^2 image at the start (a) and end (b) of the experiment with the smaller image frame measurements performed between the two. The increase in vacancy islands (VIs) throughout the experiment is evident and shows increase in the size, and number of the VIs with multiple coalescence events. Unique to this series is the depth of the VIs in the vicinity of the bunched steps labeled K1 (Figure S1-1(a)): a VI at least 5 layers deep develops in its vicinity. The "stacking depth" in this VI is unusual – most VIs on a terrace are only one or two layers deep with sequential removal of layers. This is indicative of an easier nucleation of VIs close to multi-layer step edges.

Figure S1-2 shows several images (a) to (e) extracted from an image stack of 17 images included in the GIF file in Figure S1-3. From Figure S1-2(a) to (b) nearly a complete TaS_2 layer is removed around the feature B2 (sub-surface defect) after coalescence of several islands. The VI then grows quickly and the image drift now allows us to see the increase in step bunching around B1 and K1 (see legend for features in the figure). Continued imaging leads to rapid increase in the step bunch slope through reduction in the associated terrace width: etch rates along the step edges is high, and numerous VIs nucleate close to the step bunch. This leads to a deep trench which is seen in Figure S1-1(b) after the increase of the images size to capture the larger region around the step bunch.

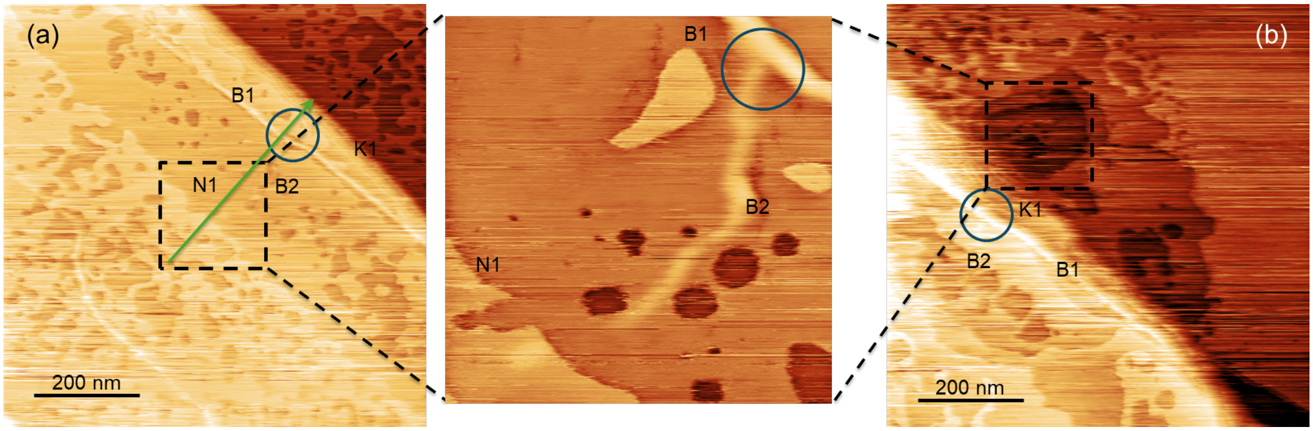

Figure S1-1: (a) STM image that shows large numbers of VIs formed in the first hour of the experiment scanned at larger image size. The black dashed square indicates the subsequent scan area after the scanner size was changed to 200×200 nm^2. (V_{bias} = 1 V, I = 50 pA). (b) After returning to 800×800 nm^2 scan area – the square indicates the position of the smaller image frame at the end of the sequence. Labels: *B1 and B2:* sub-surface defect lines, *N1:* notch in step edge, *K1:* bunched steps; *Broken lines:* (a) First image frame position, and (b) last image frame position; *Green arrow:* direction of the thermal scanner drift; *Blue circle:* branching point between B1 and B2 used to measure drift.

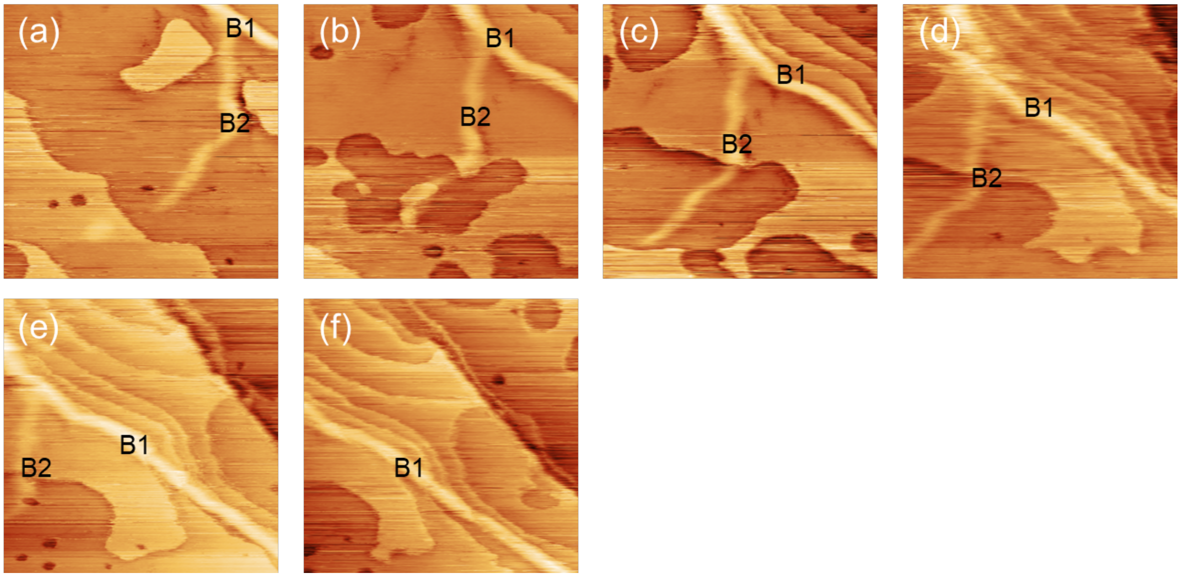

Figure S1-2: (a) to (f): Select images with 200×200 nm^2 (V_{bias} = 1 V, I = 50 pA) with increasing etch time. The image drift can be recognized by following the distinct image features B1 and B2. The complete set of 17 images in this sequence is captured in a GIF file added as Figure S1-3.

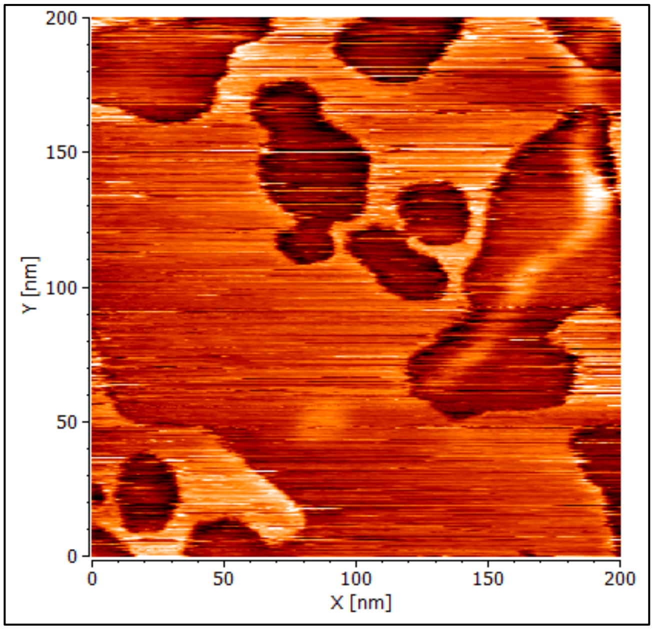

Figure S1-3: GIF movie made of 17 sequential images spanning ~144 minutes with about 500 s per image. (V_{bias} = 1 V, I = 50 pA)

S2 Test participation of scanning probe tip in the etching

To test that the STM probe, respectively the STM tip, actively participates in the etching mechanism. we carried out several scanner position changes after a few scans and observed the boundary created by the scanner. The VIs above the boundary have grown much larger in size compared to the ones below the boundary which are only captured after changing the scan region. This proves that the participation of the STM tips is essential for the surface etching discussed in this work. In addition, we held the STM tip stationary with feedback loop on in the middle of a scan and resumed after ~30 minutes, and no notable etching rate changes were discovered between the two image sections before and after the interruption. In conclusion, the scanning of the probe is essential to the etching mechanism.

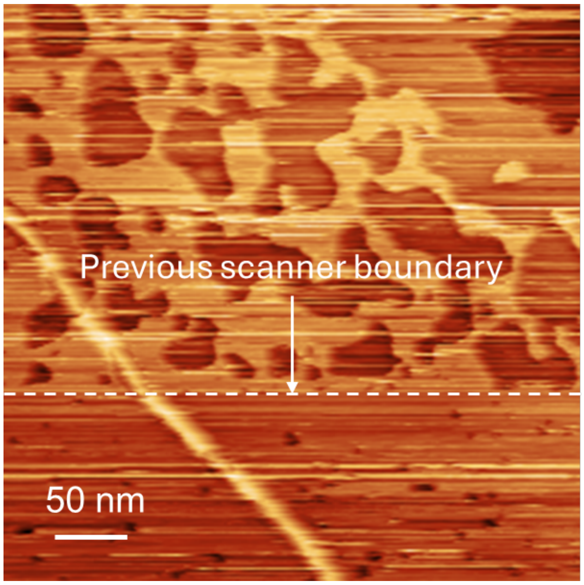

Figure S2. STM image showing the dependency of surface etching on scan region. The area upwards of the "previous scanner boundary" was scanned for ~ 2 hours, and the scan area was then moved to include the boundary to illustrate the difference. It is evident that the nucleation and growth of VIs is tip induced. The bright line extending across the image on the left-hand side is a sub-surface linear defect discussed in the main body of the manuscript.

S3 Pinning of VIs at sub-surface features

In Figure S3, we show a "pinning" effect where the growth of VIs is influenced by the sub-surface linear defects similar to the ones shown in Figure 2(e) in the main body of the manuscript and Figure S2. The sub-surface line defect in image S3 nearly bisects the image from top to bottom and is marked with a

yellow arrow. With the presence of sub-surface defects, the removal of surface TaS_2 or the growth of VIs tends to follow the pattern set by the sub-surface features or sub-surface linear defects. The removal of material in the middle of the image where the VI intersects with the sub-surface defect was delayed compared to other parts of the VI. Image S3 is a time series recorded over ~24 h, and in the first 4 image slices the top TaS_2 layer is completely removed, in the following three image slices the subsequent layer is etched. After several layers of exfoliation by the tip etching, the sub-surface line features are still visible, indicating a vertical propagation of defects during the growth process extending through several layers of the TMD. This means that once a line-type defect has formed the growth will be modified in its vicinity as the crystal growth continues. The removal of multiple layers by tip etching therefore gives us a clear image of what is happening during the CVT growth process itself and the etching serves like a local depth probe. Locations directly above the line defect remain the most active etching sites due to the structural and electronic inhomogeneities that disrupted the growth of the TMD and nucleation of subsequent layers.

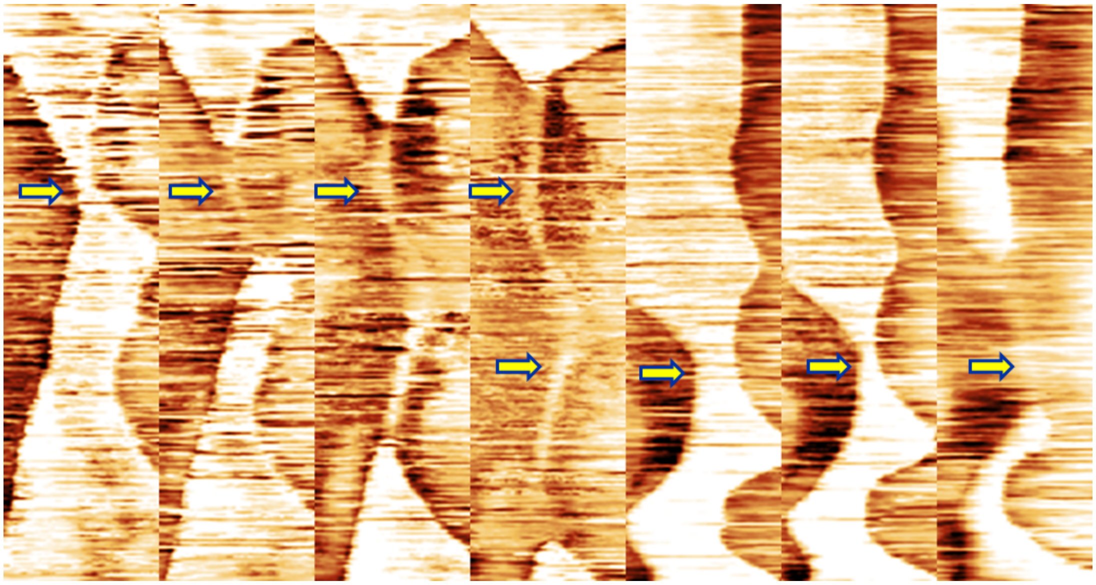

Figure S3. Sequential STM images over the same area recorded for ~ 24 hours. Different time interval between each frame. The yellow arrow indicates where the removal of the top layer is slowed/pinned by the underlying sub-surface defects.

S4 Image stack "movies" of the surface etching

To better display the progress of VI growth, we combined the images presented in this work into animated GIF movies, shown in Fig. S4-1 and S4-2. Images from these series were used to follow select VIs during their lifetime and are discussed in detail in the manuscript. It is evident that image quality varies dramatically over the course of etching and image acquisition, which we attribute to changes in the tip quality, and the propensity of the tip to pick up small TaS_2 fragments as shown in Figure 6 in the main body of the manuscript. During the imaging for the first GIF movie shown in Figure S4-1 for series 9-4 to 9-71 the imaging conditions were changed repeatedly to test the impact of the voltage and tunneling current on the etching rate. Those conditions are summarized in Tables S1 and S2. Figure S4-3 summarizes the VI growth rates for all islands with the bias voltage switch listed. Only VI#5 where the bias voltage was switched from 0.5V to 1.5V shows a change in etch rate which decreases by about 10% when switching from 0.5 to 1.5 V. This variation might still be within the error bar of the experiment and island analysis. It is worth noting that the overall etching rate is not always stable even without modulation of the bias voltage, and the role of bias voltage cannot fully be separate from other factors.

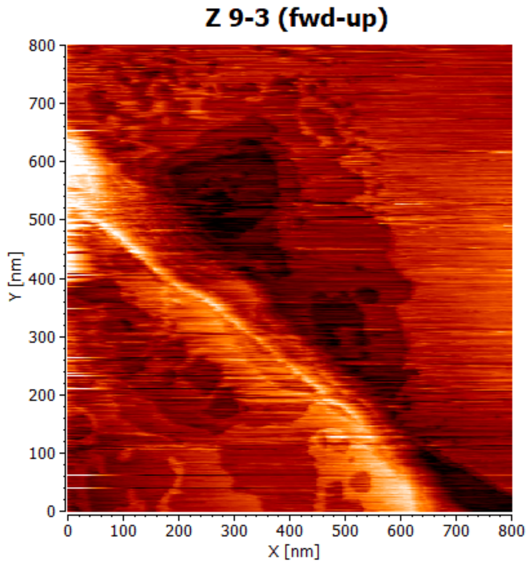

Figure S4-1. GIF movie made of 68 sequential images spanning ~19.5 hours. Only one scanning direction (up and forward) was included to avoid feature twitching.

Table S1. VIs isolated and discussed in the main body of the manuscript from *set 9 movie* and their imaging conditions. "Number" refers to the respective label used in the graphs in Figures 3, 4 and 5, and "image number" is the subsequence isolated for the analysis of the individual VIs in this set.

Vacancy Island Number	Image Number (experiment labels)	Bias (V_{bias})	Set Point (pA)	Probe Speed (nm/s)
**1**	9-20 to 9-26	0.5	20	1600
**2**	9-15 to 9-21	-1 and 0.5 (switch at 9-16)	20	1600
**3**	9-21 to 9-25	0.5	20	1600
**4**	9-49 to 9-71	0.5 and 1.5 (switch at 9-59)	20	1600
**5**	9-4 to 9-25	-1 and 0.5 (switch at 9-16)	20	1600
**Coalescence**	9-13 to 9-19	-1 and 0.5 (switch at 9-16)	20	1600

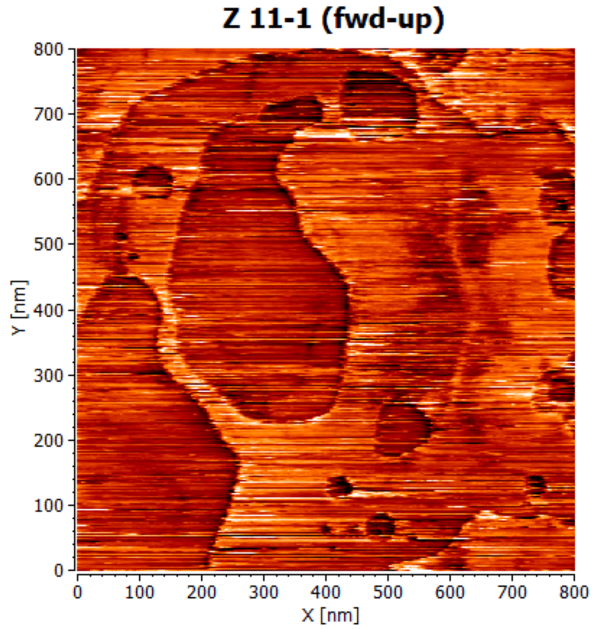

Figure S4-2. GIF movie made of 47 sequential images spanning ~13.3 hours. This series corresponds to the complete Series 11 for VIs#6 to 8.

Table S2. VIs analyzed in set 11 movie and their imaging conditions.

Vacancy Island Number	Image Number (experiment labels)	Bias (V)	Set Point (pA)	Probe Speed (nm/s)
**6**	11-3 to 11-9	1	50	1600
**7**	11-10 to 11-45	1	50	1600
**8**	11-26 to 11-45	1	50	1600

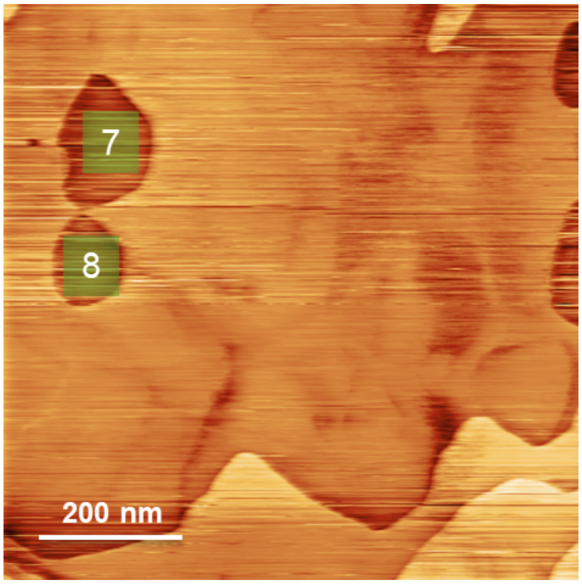

Figure S4-3. A frame from the movie Series 11, showing the position of VI #7 and #8.

S5 VI outline process to measure VI dimensions

The image masking of the VIs outlines was carried out initially by hand, and another method was chosen to extract VIs features and compared to the manual approach and measurements done in FIJI[1] for benchmarking. Selected plane-leveled images were plugged into a new AI imaging tool "Segment Anything" demo[2] to mask features through computer-vision method. The masked images were also measured in FIJI[1] for area and perimeter. We found that the difference between the area calculated from the hand draw outline and Segment Anything is <3%. The challenges remain that very streaky images cannot be masked reliably despite the fact that they are still visible to human inspection. However, even the hand drawn outlines become error prone, and we therefore limited our analysis to those VIs with the smallest errors.

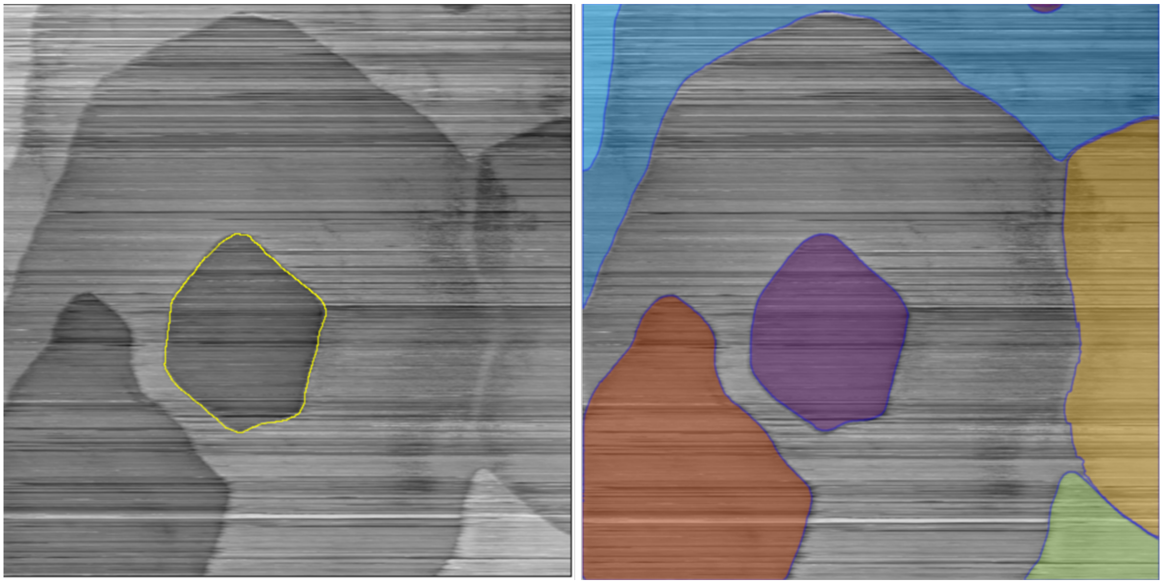

Figure S5. A comparison between the two methods used for masking VIs: (Left) Outlining by hand in FIJI [1] (Right) Auto-masking through Segment Anything demo.[2]

S6 Screw dislocation

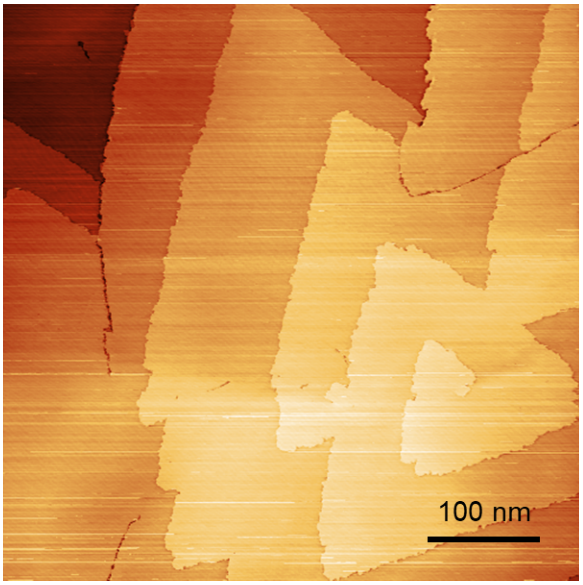

Figure S6. STM image of a separated region enriched with topological defects, including a screw dislocation, found on the 2H-TaS_2.

Fig. S8 shows a screw dislocation that has a Euclidean surface and aligned spiral angle.[3] Screw dislocations can be typical to the sample grown by the chemical vapor methods. The curvature of the vertexes is round instead of pointy, which is expected in the stacking of the 2H prototype layered matierlas.

S7 Removal of TaS_2 in vicinity of surface line defects

Figure S9 shows an example of VI formation and growth as it is initiated at the boundaries of large line defects. These defects, most likely related to the use of a flux agent during CVT growth, are seen clearly in Figure 1 of the manuscript. The rate of etching could not be determined since many of the VIs are linked and undergo many coalescence events but it is evident that the perimeter of the line defects serves as nucleation site for VIs.

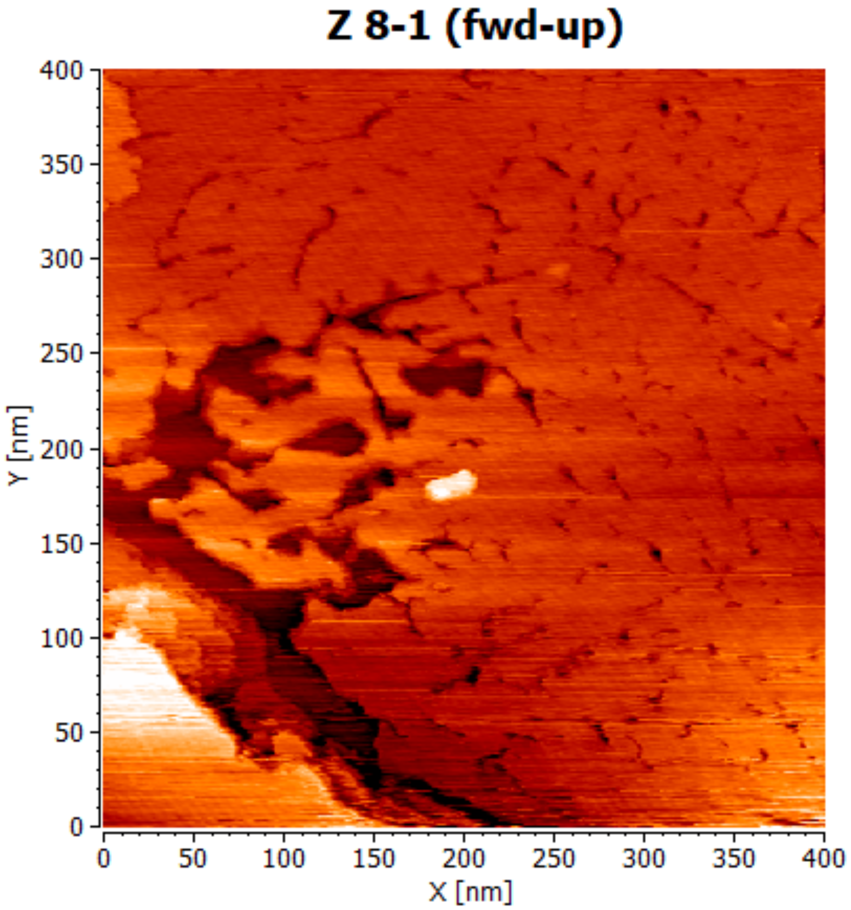

Figure S7: Tip induced etching, nucleation and growth of VIs starting from the boundaries of line defects.

References

(1) Schindelin, J.; Arganda-Carreras, I.; Frise, E.; Kaynig, V.; Longair, M.; Pietzsch, T.; Preibisch, S.; Rueden, C.; Saalfeld, S.; Schmid, B.; et al. Fiji: an open-source platform for biological-image analysis. *Nat Methods* **2012**, *9* (7), 676-682. DOI: 10.1038/Nmeth.2019.
(2) Kirillov, A.; Mintun, E.; Ravi, N.; Mao, H.; Rolland, C.; Gustafson, L.; Xiao, T.; Whitehead, S.; Berg, A. C.; Lo, W.-Y.; et al. Segment Anything. *arXiv e-prints* **2023**, arXiv:2304.02643. DOI: 10.48550/arXiv.2304.02643.
(3) Zhao, Y. Z.; Jin, S. Stacking and Twisting of Layered Materials Enabled by Screw Dislocations and Non-Euclidean Surfaces. *Accounts Mater Res* **2022**, *3* (3), 369-378. DOI: 10.1021/accountsmr.1c00245.
